# Therapeutic Effects of *Hab Shabyar* on Open-Angle Glaucoma: A Double-Blinded, Randomized, Placebo-Controlled Trial

**DOI:** 10.31661/gmj.v9i0.1218

**Published:** 2019-01-01

**Authors:** Ebrahim Khalil- BaniHabib, Ali Mostafai, Seyyed Mohammad Bagher Fazljou, Ghadir Mohammdi

**Affiliations:** ^1^Faculty of Traditional Medicine, Tabriz University of Medical Sciences, Tabriz, Iran; ^2^Research Center for Evidence-based Medicine, Ophthalmology Department, Tabriz University of Medical Sciences, Tabriz, Iran

**Keywords:** Glaucoma, Open Angle, Hab Shabyar, Timolol

## Abstract

**Background:**

Open-angle glaucoma (OAG) is one of the leading causes of blindness worldwide. This study evaluates the therapeutic effects of *hab shabyar* in patients with open-angle glaucoma.

**Materials and Methods:**

In this clinical randomized controlled trial, 50 patients with OAG were randomized into two groups. The intervention group received a drop of timolol plus 500 mg of *hab shabyar* every 12 hours. The placebo group received a drop of timolol every 12 hours plus 500 mg of wheat germ as a placebo. The intraocular pressure in patients with OAG was measured in each group and compared before the intervention (t1), one month (t2), and two months (t3) after the intervention.

**Results:**

The mean decrease in intraocular pressure for the right eye on three times in the intervention group was statistically significant, but the mean decrease in the placebo group was not significant. Similar results were obtained for the left eye at t1 when compared to t3. The intervention group patients expressed more satisfaction changes than the placebo group (P≤0.001).

**Conclusion:**

Our study demonstrated that consumption of timolol plus *hab shabyar* instead of consuming timolol alone was probably more effective for reducing intraocular pressure in patients with OAG.

## Introduction

Open-angle glaucoma (OAG) is one of the most common types of glaucoma and one of the most common causes of blindness worldwide [1]. Glaucoma is determined by increasing the eye’s internal pressure to deepen the optic disc and visual disturbance. In OAG, despite the openness in the anterior chamber angle, the outlet speed of aqueous humor from the anterior chamber is low, which causes this fluid to accumulate and the pressure inside the eye to increase. This increase in intraocular pressure causes damage to the optic nerve [2]. OAG accounts for approximately 74% of glaucoma cases globally [3]. The prevalence of OAG has been reported for most provinces of Iran; for instance, it is 1.33% in Tehran [4], 1.44% among 14-year-olds [5], 2.9% in Khozestan province [6], and 4.4% in Yazd [7]. The prevalence of OAG worldwide also varies; for example, it is 3.9% among all age groups in Japan [8], 1.8% in Australia [8], and 4.74% in California [9]. The etiology of OAG is multifactorial and is likely influenced by environmental and genetic factors. Some risk factors have an established association with OAG, including African ethnicity, family history of glaucoma, older age, and elevated intraocular pressure [10]. On the other hand, the associations between OAG and smoking, hypertension, diabetes mellitus, socioeconomic background, nutritional supplements, and gynecological factors remain controversial [11,12]. In the treatment of glaucoma, topical beta-adrenergic and alpha-adrenergic agonists, topical and systemic carbonic anhydrase inhibitors are used to prevent the production of aqueous fluids, and para-sympathomimetic drugs and prostaglandin analogs are used to facilitate the removal of this liquid. In the absence of response to drug therapy, trabeculectomy and laser trabeculoplasty are used as surgical treatments [13]. The use of herbal remedies in traditional medicine has a long history because of their low cost, few side effects in comparison to chemical drugs, availability, and efficacy [14]. In Persian medicine, statements have been made about treating increased aqueous humor and the presence of obstruction at its outlet, which causes reduced vision and even blindness [15]. For{Avaz-e-kermanI, 2014 #2} this purpose, some traditional medicine books have proposed treating glaucoma with *hab shabyar* [16]. The original combination of *hab shabyar* is with *Aloe vera* [17]. *Hab shabyar* is combined with compounds such as *Aloe vera*, *Terminalia chebula*, *Citrullus colocynthis*, *Pictacia lentiscus*, valerian, *Origanum vulgare*, *Rose centifolia*, *Crocus sativus*, and foam of honey [18]. As we know, to produce the highest level of evidence among primary studies, clinical randomized controlled studies are the best and gold standard in clinical medicine and public health for evaluating the efficacy and side effects of new therapeutic or preventive interventions [19]. Randomized controlled trials have an essential role in identifying the adverse effects of relatively common therapies and those that occur relatively soon after the therapy has been initiated [20]. Therefore, considering that no randomized controlled trial to date has confirmed the effect of *hab shabyar* in the treatment of OAG, the present study was conducted to assess the efficiency of timolol plus *hab shabyar* compared to timolol alone (as a routine drug) in treating patients with OAG who were referred to clinics and educational, therapeutic hospitals affiliated with Tabriz University of Medical Sciences.

## Materials and Methods

###  Design and Setting

 This double-blind, randomized, placebo-controlled trial was conducted on patients with OAG and eye pressure of 12-21 mmHg who were referred to clinics and educational-therapeutic hospitals affiliated with Tabriz University of Medical Sciences between May 2016 and March 2017. Ethical approval (IR.TBZMED.REC.1395.9) was obtained from the centralized institutional review board of Tabriz University of Medical Sciences; then, the study was registered on the Iranian Registry of Clinical Trials (registration code: IRCT2016052828127N1). Also, written informed consent was obtained from all participants.

###  Sample Size Calculation and Randomization

 The sample size for this study was calculated by the difference in means of two-eye pressure in the intervention (24.0 ± 2.10) and placebo (22.0 ± 2.2) groups based on statistics from other studies, statistical power of 80%, and a confidence level of 95%. The sample size formulas [n = (zα/2 + zβ)2 * (s21 + s22) / (x̅1 − x̅2)2] for each group obtained 17 people. In the end, taking into account a 20% loss of participants and 25 samples for each group, 50 samples were included. A total of 50 patients with OAG were allocated into two groups (n=25). The random allocation sequence based on the random number table. Briefly, we first closed our eyes and then placed one of the numbers on the table and recorded the house number and the number at the starting point. We also noted the direction of the move. The group assignment of eligible items was not known. In this study, all patients with odd numbers were assigned to the intervention group, and all patients with an even number were assigned to the comparison group.

###  Inclusion and Exclusion Criteria

 The inclusion criteria in this study were aged between 20 to 70 years old, cases of OAG with 12-21 mmHg intraocular pressure, and patient approval for participating in the trial. Patients with closed-angle glaucoma, pregnancy, Crohn’s disease or ulcerative colitis, and continuously consumed intraocular pressure drugs were excluded.

###  Data Collection

An interviewer-administered questionnaire was used to collect data on characteristics such as gender, education, the region of residence, and duration of morbidity. Then, all patients underwent a comprehensive ophthalmic examination. OAG was defined by glaucomatous optic neuropathy (cup-disc ratio of >0.7 or an inter-eye asymmetry of >0.2 and/or glaucomatous notching) with compatible visual field loss and open angles on gonioscopy. The intervention group was given a drop of timolol every 12 hours plus 500 mg *hab Shabyar* (once-daily, orally) before sleep. The placebo group was given a drop of timolol every 12 hours plus 500 mg wheat germ as a placebo (once daily, orally) before sleep. After three weeks, both groups’ intraocular pressure was evaluated using a Goldmann tonometer (Haag-Streit AT 900, Switzerland). Intraocular pressure in the patients in each group was measured three times: before the intervention (t1), one month (t2), and two months (t3) after the intervention. Finally, the changes in the mean intraocular pressure in both the intervention and placebo groups were compared.

###  Statistical Analysis

 Statistical analyses were conducted using the statistical software Statistical Package for Social Science (SPSS, Version 20; IBM Corp., Armonk, NY, USA). Descriptive statistics were computed for all variables, including means for the continuous variables, frequencies for the categorical variables, and standard errors of the means. A t-test was used to compare the means. A chi-square test was used to determine the frequency distributions in the intervention and placebo groups. Repeated measures were used as a statistical test to compare the effects of treatment on the intraocular pressure at three times. P-value<0.05 was considered as the statistical significance level.

## Results

**Figure 1:**
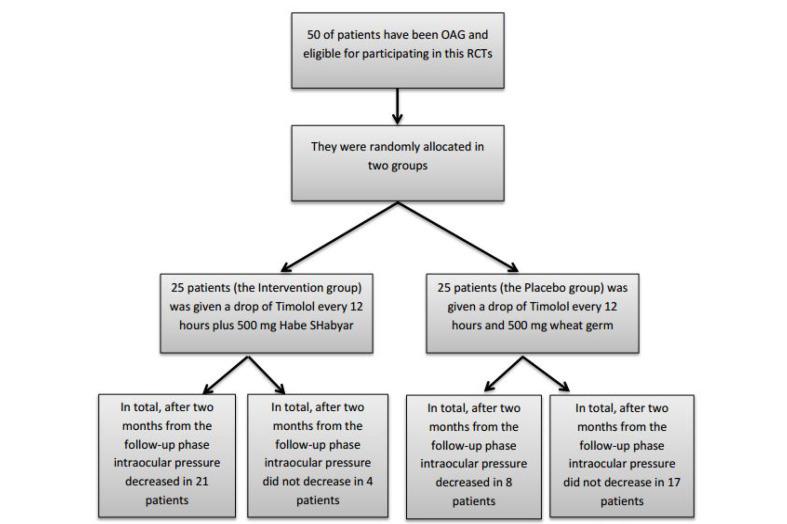
CONSORT flow diagram of studied patients with OAG ^CONSORT flow diagram of studied patients with OAG^

 In this study, 50 confirmed cases with OAG were randomly allocated to the intervention and placebo groups. As shown in Figure-1, there were no losses or exclusions after randomization. The distributions of demographic characteristics among patients with OAG in both the intervention and placebo groups are summarized in Table-1. The results obtained from Table-2 show much more impressive trends regarding reduced intraocular pressure in both the eyes of OAG patients who received the interventional treatment than those who received a placebo. As shown in Table-3, the mean decrease in intraocular pressure for the right eye at three times (t1 in comparison with t2, t1 in comparison with t3, and t2 in comparison with t3) in the intervention group was statistically significant, but in the placebo group was not significant at the three mentioned times. Similar results were obtained for the left eye in t1 compared to t3, as shown in Table-4. Patients’ satisfaction and complications during treatment in the intervention and placebo groups are presented in Table-5.

## Discussion

The prevalence of glaucoma is increasing in both developed and developing countries [21]. Some traditional medicine books have proposed *hab shabyar* to treat glaucoma [16]. This study mainly focuses on detecting the therapeutic effects of *hab shabyar* in patients with OAG. Indeed, it is novel and the first randomized clinical trial study to evaluate the therapeutic effects of *hab shabyar* in patients with OAG. One of the remarkable findings in this study was the beneficial effect of simultaneous consumption of *hab shabyar* and timolol in reducing the mean intraocular pressure in patients with OAG compared with timolol alone. However, it should be noted that the mechanism of this intraocular pressure reduction is only somewhat known. *Hab shabyar* could dry aqueous humor, and it is also capable of cleaning and restoring the brain as well as removing obstructions in the outlet of aqueous humor [17]. Janet * et al.,* in a randomized, double-blinded trial in 2017, reported that once-daily dosing of netarsudil to timolol 0.02% was found effective and well-tolerated for the treatment of patients with ocular hypertension and OAG [22]. Whitcup *et al*. [23] reported that bimatoprost 0.03% once daily demonstrated superior efficacy compared with timolol 0.5% twice daily in patients with elevated intraocular pressure. Also, bimatoprost once daily was more effective than twice-daily dosing. Ozer *et al*. [24] found that brimonidine+ timolol, dorzolamide+timolol, and latanoprost+timolol combination therapies showed similar lowering efficacies on OAG levels, whereas there was no any difference between each other. To identify the pharmacological mechanisms and the synergistic interaction between *hab shabyar* and timolol, they must be investigated in specific and separate studies. Variables such as gender, education, region, and duration of morbidity distort *hab shabyar* and timolol’s effectiveness in reducing the mean intraocular pressure in patients with OAG. There were no any significant differences between patients with OAG who were randomly assigned to the intervention and placebo groups. The homogeneity of characteristics among the patients in the two groups increased the internal validity of this study. As we have already stated, one of the leading combinations for *hab shabyar* is with *Aloe vera*, and its pharmacological attributes have been evaluated in modern science to prove that the drug has immense potential in pharmacotherapeutics [25]. Our data are consistent with an emerging study showing that *Aloe vera* effectively treated glaucoma [17]. For instance, Son et al. mentioned that after consumption of *Aloe vera* had reduced glaucoma at least 90% of their new cases [26]. A similar study conducted by Serduyk *et al*. in line with our finding reported that *Aloe vera* could reduce the primary OAG [27]. On the other hand, several biological activities of *Aloe vera* as herbal remedies have been studied. For example, Saberi *et al*. [28]. revealed that *Aloe vera* was useful for improving the thickness of the retina and its layers retained their normal histologic structures. *Aloe vera* also has long been used as a traditional medicine to promote wound healing, anti-inflammatory, antifungal activity, anticancer and immunomodulatory. In general, it is a natural product that nowadays is used in the cosmetic industry [25]. Our results indicate a significant difference between the intervention and placebo groups in terms of satisfaction with their consumption of drugs. Indeed, 64% and 0% of patients with OAG in the intervention and placebo groups declared that they were delighted with their drug use, respectively. On the other hand, only 16% of patients with OAG in the intervention group declared that they were dissatisfied with their drug use, respectively. There was no any significant difference in the complications of each treatment among the intervention and placebo groups. The present study has a few limitations. Firstly, our study participants included patients with OAG who were referred to clinics and educational-therapeutic hospitals affiliated with the Tabriz University of Medical Sciences. Some characteristics of the patients with OAG in the present study may be substantially different from those of other populations. Therefore, the external validity of our study may be limited. Secondly, there was limited data available on the family history of glaucoma among the patients under study; thus, this variable had to be excluded from the analysis. Further research should consider this variable in examining this relationship between timolol and *hab shabyar*.

## Conclusion

Our finding suggests that the consumption of timolol plus *hab shabyar* instead of timolol alone is probably more useful for reducing the intraocular pressure in patients with OAG. Then, this evidence could be useful for national prevention programs for blindness and low vision to achieve the VISION 2020 goals and the right to sight.

## Acknowledgment

 This study was driven by a Ph.D. thesis in traditional medicine. This research project has been funded by a grant (1395.9) awarded by the Tabriz University of Medical Sciences. The authors would like to thank all those who supported us and participated in this study.

## Conflicts of Interest

 None to declare.

**Table 1:** Distribution of Demographic Characteristics Among Patients With OAG in the Intervention and Placebo Groups

**Variables**	**Subgroups**	**Intervention group** **n (%)**	**Placebo group** **n (%)**	**P-value**
**Gender**	Male	14 (56)	13 (52)	0.571
	Female	11 (44)	12 (48)	
**Education**	Illiterate	10 (40)	13 (52)	0.142
	Primary school	3 (12)	3 (12)	
	Middle or high school	3 (12)	1 (4)	
	Diploma	6 (24)	4 (16)	
	Associate’s degree	2 (8)	0 (0)	
	Bachelor’s degree	1 (4)	4 (16)	
**Region of residence**	Rural	0 (0)	4 (16)	0.11
	Urban	25 (100)	21 (84)	
**Duration of morbidity, y**	≤1	6 (24)	6 (24)	0.565
	2-5	10 (40)	11 (44)	
	6-9	7 (28)	5 (20)	
	≥10	2 (8)	3 (12)	

**Table 2:** Means of Right and Left Eyes Pressure Based on Treatment. Data Are Presented As Mean ± SD.

**Time**	**Right eye**		**Left eye**	
	**Intervention group**	**Placebo group**	**Intervention group**	**Placebo group**
**t1** **(95% CI)**	21.52 ± 5.09 (19.42, 23.62)	17.92 ± 12.04 (12.95, 22.89)	18.08 ± 7.09 (15.15, 21.01)	16.60 ± 3.94 (14.97, 18.23)
**t2** **(95% CI)**	17.76 ± 4.16 (1.2 ± 36.19)	18.32 ± 12.07 (13.34, 23.30)	15.88 ± 6.53 (13.19, 18.57)	16.64 ± 3.85 (15.05, 18.23)
**t3** **(95% CI)**	16.16 ± 3.93 (14.54, 17.78)	18.96 ± 11.97 (14.02, 23.90)	14.96 ± 6.23 (12.39, 17.53)	17.68 ± 4.19 (15.95, 19.95)

**t1**: before the intervention; **t2**: one month after the intervention; **t3**: two months after the intervention; **CI**: Confidence interval

**Table 3:** Mean Intraocular Pressure in the Right Eye at Different Times in the Intervention and Placebo Groups

**Time**	**Intervention group**			**Placebo group**		
	**Mean difference**	**SD **	**P-value**	**Mean difference**	**SD **	**P-value**
**t**_1 _ **compared to t**_2_	3.76	0.95	0.002^*^	−0.40	0.63	0.762
**t**_1 _ **compared to t**_3_	5.36	1.01	<0.001^**^	−1.04	0.76	0.549
**t**_2 _ **compared to t**_3_	1.60	0.48	0.008^*^	−0.64	0.76	0.97

*Significant at the 0.05 level (2-tailed) **Significant at the 0.01 level (2-tailed)

**Table 4:** Mean Intraocular Pressure in the Left Eye at Different Times in the Intervention and Placebo Groups

**Time**	**Intervention group**			**Placebo group**		
	**Mean difference**	**SD **	**P-value**	**Mean difference**	**SD**	**P-value**
**t**_1 _ **compared to t**_2_	2.20	0.88	0.06	−0.004	0.34	0.865
**t**_1 _ **compared to t**_3_	3.12	0.87	0.004^*^	−1.08	0.62	0.285
**t**_2 _ **compared to t**_3_	0.92	0.39	0.082	−1.04	0.62	0.316

*Significant at the 0.05 level (2-tailed)

**Table 5:** Distribution of Patients’ Satisfaction and Treatment Complications Among the Intervention and Placebo Groups

**Variables**	**Intervention group ** **(n = 25)**	**Placebo group** ** (n = 25)**	**P-value**
**Patients’ satisfaction, n (%)**			
Very satisfied	16 (64)	0 (0)	< 0.001
Satisfied	9 (36)	21 (84)	
Dissatisfied	0 (0)	4 (16)	
**Complications of Treatment, n (%)**			
Nausea	0 (0)	1 (4)	0.442
Diarrhea	1 (4)	0 (0)	
Other	2 (8)	2 (8)	
No complications	22 (88)	22 (88)
